# The effects of exercise therapy on the prognosis of patients with COVID-19: A protocol for systematic review

**DOI:** 10.1097/MD.0000000000023762

**Published:** 2020-12-18

**Authors:** Guorong Qiu, Yu Ji, Yajun Tan, Benxiang He, Chunfeng Tan, Zhuoling Wang, Hongpan Gao

**Affiliations:** a School of Sports Medicine and Health, Chengdu Sport University, No. 19 West Section of 1st Ring Road, Chengdu, Sichuan Province; b Institute of Sports History, Chengdu Sport University, Chengdu; c Sport Hospital Attached to Chengdu Sport University, No.19 West Section of 1st Ring Road, Chengdu, Sichuan Province, China.

**Keywords:** Corona Virus Disease 2019, exercise therapy, systematic review

## Abstract

**Background:**

Since the COVID-19 outbreak in 2020, more than 20 million people worldwide have been diagnosed with COVID-19, and all health care workers are looking for ways to improve the cure rate of the disease. As an important method of rehabilitation therapy, exercise therapy has been proved to improve the level of human function, promote the recovery of diseases, and improve the human immune ability. The main objective of this study was to provide reliable methods and credible evidence for exercise therapy to improve the prognosis of COVID-19 patients.

**Methods and analysis:**

The scheme was systematically reviewed in accordance with the preferred reporting items. We searched the following experimental databases: Cochrane Library, PubMed, EMBASE, Web of Science, China Biomedical Literature Database (CBM), China National Knowledge Infrastructure Database (CNKI), China Science and Wanfang Database. All trials using exercise therapy for rehabilitation of COVID-19 patients in the above database should be considered for inclusion. Relevant randomised controlled trials(RCTS), controlled before and after, interrupted time series and prospective analytic cohort studies regardless of publication date, language and geographic location, will be included. To summarize the therapeutic effect of exercise therapy on COVID-19 patients, high-quality literature was selected for data extraction and analysis. Two reviewers will independently screen titles, abstracts and full-text articles against inclusion criteria; perform data extraction and assess risk of bias in included studies. We will assess the certainty of the overall evidence using the Grading of Recommendations Assessment, Development and Evaluation approach and report findings accordingly.

**Results:**

In this study, we hope to summarize effective exercise therapy that can improve the prognosis of COVID-19 patients and find strong evidence for it.

**Conclusions:**

The conclusions of this study will provide reliable evidence to determine whether exercise and exercise therapy can improve the prognosis of COVID-19 patients and guide future studies.

**PROSPERO registration number:**

CRD42020209025.

## 1 Introduction

At the end of 2019, a pneumonia caused by a new pneumonia virus declares its fast spread throughout the World, and thus brings a great impact on global public Health, Health care system and economic development.^[1]^ On February 7, 2020, the World Health Organization (WHO) designated COVID-19 as Corona Virus Disease 2019 (COVID-19).^[2]^ Genome sequencing has confirmed that COVID-19 is similar to SARS-CoV and bat coronavirus, but the main source, mode of transmission, and mechanisms associated with human pathogenicity of this pandemic outbreak are unknown.^[3]^ At the beginning, COVID-19 is characterized by fever, dry cough, fatigue, and then dyspnea. In severe cases, it may progress to acute respiratory distress syndrome, septic shock, or even death.^[4]^ Pneumonia often affects both lungs and presents mainly in the lower lobe, it is more severe in elderly patients over 70 years of age and in patients with various complications.^[5]^ Most children and young adults with SARS-COV-2 develop mild to moderate influenza-like symptoms and have a good prognosis. In addition to respiratory symptoms, some patients also experience digestive problems such as vomiting and diarrhea.^[6]^ The transmission mode of the virus is to enter the human body through mucous membranes, especially the mucous membranes of the nose and throat, and reach the lungs through the respiratory tract, and quickly replicate and spread.^[7]^ virus can be asymptomatic infection close (2 meters) cough or sneeze droplets spread, this is the main way for the spread of the virus. The fecal-oral route may also be a route of infection, as tests have shown that the virus is present in the feces and urine of COVID-19 patients.^[8,9]^ The main treatment for COVID-19 is symptomatic treatment, with critical patients receiving organ support. The use of drugs by antiviral drugs,^[10]^ protease inhibitors^[11]^ and a variety of drugs combined application,^[12]^ the purpose is to reduce the patient's clinical symptoms. Traditional Chinese medicine (TCM) has also been widely used in COVID-19 patients in China. In clinical treatment, combined with conventional symptomatic treatment, good therapeutic effect has been achieved.^[13]^

Exercise therapy refers to the use of equipment, freehand or patient's own strength, exercise therapy through some means of movement (active or passive movement, etc.), so that patients get the whole body or local motor function, sensory function recovery training methods. Long-term outcomes for COVID-19 are not fully understood. Based on existing clinical studies, the possible results can be briefly categorized as follows:

1. Lung injury that may develop, especially in severe cases of pneumonia or acute respiratory distress syndrome (ARDS);^[14]^
2. Complications during intensive care, such as fixation;
3. Possible consequences and complications of neurological symptoms or disease caused by COVID-19;
4. Lack of exercise, changes in eating habits, insomnia and psychological problems during isolation or isolation.^[15]^

For patient rehabilitation exercise and abdominal breathing training exercise therapy can effectively increase the patient breathe out capacity, improve lung function.^[16]^ Through training exercise therapy in patients with upper limb muscles, lower limb muscles, walking training, breathing training, etc., can reduce patients with dyspnea condition, improve the patients’ pulmonary ventilation, pulmonary ventilation function, reduce the patients’ sputum, cough, improve function of limbs movement, make the patients life self-care ability,^[17]^ the effect of COVID - 19 has a benign prognosis.

## 2 Method

### 2.1 The registration

In accordance with the guidelines, this systematic review protocol was registered with the International Prospective Register of Systematic Reviews (PROSPERO) on September 15, 2020 (registration number CRD42020209025).

### 2.2 Inclusion criteria for study selection

#### 2.2.1 Study designs

We will include researches related to exercise therapy of patients suffering from COVID-19. Due to language restrictions, we will search for articles in English and Chinese. In order to get a more objective and true evaluation, all articles must meet the following four conditions at the same time:

(1) Published documents with complete documents data;
(2) Participants were confirmed to have COVID-19;
(3) The intervention group received exercise therapy intervention for at least a period of treatment.
(4) We will include randomized controlled trials (RCTs), controlled (non-randomized) clinical trials (CCTs) or cluster trials, controlled before-after (CBA) studies, prospective and retrospective comparative cohort studies, and case-control or nested case-control studies. CBA studies will be included only if there are at least two intervention sites and 2 control sites.

We will exclude cross-sectional studies, case series, and case reports.

#### 2.2.2 Participants

We will include all patients who suffering from COVID-19 regardless of sex, age, racial group, education, and economic status. Pregnant women, postoperative infections, psychopaths, patients with severe pneumonia or other reasons who cannot exercise, patients with severe cardiovascular and/or liver and/or kidney diseases will not be included.

#### 2.2.3 Interventions

The studies at least one of the groups received exercise intervention will be included. Exercise therapy including: joint function training, muscle strength training, aerobic training, balance training, facilitation training, riding training, walking training, Ethnic form of physical therapy. Other stimulation methods such as postoperative rehabilitation training, sports rehabilitation after fracture and athlete training will be excluded.

#### 2.2.4 Comparators

The comparisons will include retreats, modern medical treatment, Traditional Chinese medicine treatment, physical factor therapy. In addition, the study will include studies comparing the use of exercise therapy in combination with another treatment method with the use of exercise therapy alone, or studies comparing the use of exercise therapy in combination with another treatment method with the use of other treatments alone.

#### 2.2.5 Outcomes

##### 2.2.5.1 Primary outcomes

1. Time of disappearance of main symptoms (including fever, asthenia, cough, Nucleic acid test,temperature recovery time) and Indicators of body function (blood pressure, heart rate, body composition, muscle strength, range of motion of the joints, Activity of Daily Living)
2. Accompanying symptoms (such as myalgia, expectoration, stuffiness, runny nose, pharyngalgia, anhelation, chest distress, dyspnea, crackles, headache, nausea, vomiting, anorexia, diarrhea) disappear rate, negative COVID-19 results rate on two consecutive occasions (not on the same day), CT image improvement, average hospitalization time, occurrence rate of common type to severe form, clinical cure rate, and mortality.

##### 2.2.5.2 Secondary outcomes

(1) Withdrawal (defined as participant withdrawal following randomization before, or during, receipt of the intervention because of consent or medical reasons).
(2) Adherence (defined as participant completion of the intervention as described in the trial methods).
(3) Mortality (defined as death at any point during the trial duration).
(4) Loss to follow-up (defined as non-completion of outcome measures due to non-attendance or other reasons, if reported).
(5) Adverse events (non-mortality).

### 2.3 Data sources

The following electronic databases will be searched from inception to June 2020: PubMed, the Cochrane Central Register of Controlled Trials (CENTRAL), EMBASE, MEDLINE, Web of Science, Traditional Chinese Medicine, China National Knowledge Infrastructure, Chinese Biomedical Literature Database, Chinese Scientific Journal Database (VIP database), and Wan-Fang Database.

### 2.4 Search strategy

The search terms on PubMed are as follows: exercise therapy (e.g., “Kinesiotherapy” or “Therapeutic Training” or “movement” or “train”); COVID-19 (e.g., “Corona Virus Disease 2019” or “Novel Corona Virus”); convalescence (e.g., “rehabilitation” or “recovery” or “decubation”); randomized controlled trial (e.g., “randomized” or “randomly” or “clinical trial”). Combinations of Medical Subject Headings (MeSH) and text words will be used. The same search term is used in other electronic databases. These search terms are shown in Table 1.

**Table 1:** Search strategy for the PubMed database.

1	exercise therapy
2	Kinesiotherapy
3	Therapeutic Training
4	movement
5	train
6	1 or 2–5
7	COVID-19
8	Corona Virus Disease 2019
9	Novel Corona Virus
10	7 or 8–9
11	convalescence
12	rehabilitation
13	recovery
14	decubation
15	11 or 12–14
16	randomized controlled trial
17	randomized
18	randomly
19	clinical trial
20	non-randomized clinical trials
21	Controlled clinical trials
22	cluster trials
23	controlled before-after studies
24	16 or 17–23
25	6 and 10 and 15 and 24

### 2.5 Data collection and analysis

#### 2.5.1 Selection of studies

**Figure 1:**
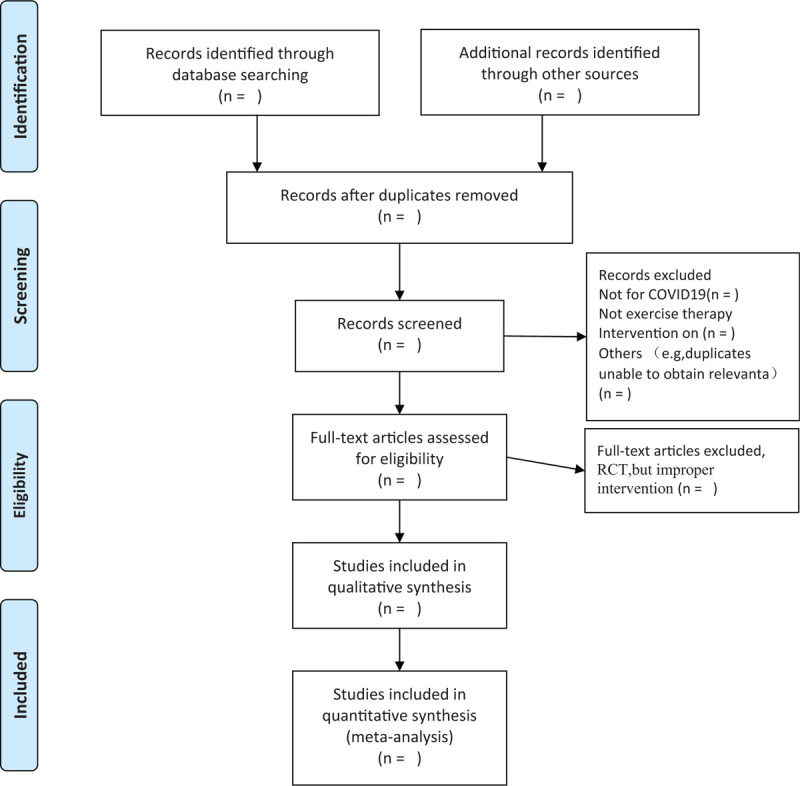
Flow chart of the study. Adapted from Preferred Reporting Items for Systematic Reviews and Meta-Analyses Protocols (PRISMA-P). COVID-19 = Corona Virus Disease 2019, RCT = Randomized controlled trial.

We chose the PRISMA flow chart to show the process of selecting literature for the entire study (Fig. 1). All reviewers will discuss and determine the screening criteria before searching the literature. Subsequently, the two review authors (YJ, YJT) will independently review and screen the titles and abstracts yielded by the search against the inclusion criteria. In order to get qualified studies, we will then screen the full text reports and decide whether these meet the inclusion criteria and then excluded some duplicate studies or studies with incomplete information. The obtained literature will be managed by using EndNote software V.X8 (Thomson Corporation, United States). Any inconsistency is resolved by discussing with the third investigator.

#### 2.5.2 Data extraction and management

We will strictly follow the inclusion criteria and select articles that are highly relevant to the topic. Through the analysis of the articles, we understand the various characteristics of the participants (such as gender, age, weight, main symptoms and duration of underlying disease, etc.), the implementation of the intervention scheme (such as the type of exercise therapy, treatment time and frequency) and results (such as the main symptoms disappear time, cure rate, severe pneumonia incidence, mortality rate, average hospitalization time), adverse reactions, bias and funding to assess risk, etc. If there is any disagreement between the two authors in the literature data extraction, a third participant will discuss the decision together. If data is lacking in the literature, we will contact the author or publisher whenever possible.

#### 2.5.3 Assessment of risk of bias in included studies

We will use the Cochrane collaborative tool to independently assess the risk of bias in the included studies. We will evaluate the following aspects of the article: sequence generation, assignment sequence hiding, blindness of participants and staff, outcome evaluators, incomplete result data, selective result reporting, and other sources of bias. The risk of bias is evaluated at 3 levels, namely, low risk, high risk, and ambiguity. If the information is vague, we will try to contact the author of the article.

#### 2.5.4 Measures of treatment effect

In this protocol, we will use 95% confidence interval (CI) risk ratio to rigorously analyze the dichotomous data. And for the continuous data, mean difference (MD) or standard MD is used to measure the efficacy of 95% CI.

#### 2.5.5 Missing data dealing with

Whenever the missing or unclear data are identified, we will contact original trial authors to request them. Otherwise, we will analyze the available data if we cannot achieve them.

#### 2.5.6 Data synthesis

Each outcome will be calculated and combined using the RevMan 5.3. Specific implementation was based on the current version of the Cochrane Handbook for Systematic Reviews of Interventions. If tests of heterogeneity are not significant, the Mantel-Haenszel method will be chosen for fixed effect model, and if statistical heterogeneity is observed (I^2^ ≥ 50% or *P* < .1), the random effects model will be used. If heterogeneity is substantial, we will perform a narrative, qualitative summary.

#### 2.5.7 Subgroup analysis

We will perform subgroup analysis according to the different details of interventions, study quality and outcome indicators.

#### 2.5.8 Sensitivity analysis

Sensitivity analysis will be performed according to sample size, study design, heterogeneous quality, methodological quality and statistical model, the trials with quality defects will be excluded to ensure the stability of the analysis results.

#### 2.5.9 Grading the quality of evidence

This paper will use the evidence quality rating method to evaluate the results obtained form this analysis. GRADE will be assessed across the domains of risk of bias, consistency, directness, precision and publication bias. In the context of the system review, quality reflects our confidence in the effectiveness of assessment. It has 4 evaluation levels, namely, high (further research is very unlikely to change our confidence in the estimate of effect), moderate (further research is likely to have an important impact on our confidence in the estimate of effect and may change the estimate), low (further research is very likely to have an important impact on our confidence in the estimate of effect and is likely to change the estimate), or very low (very uncertain about the estimate of effect).

### 2.6 Ethical review and informed consent of patients

Ethics and dissemination: The content of this article does not involve moral approval or ethical review and will be presented in print or at relevant conferences.

## 3 Discussion

Since the winter of 2019, the number of COVID-19 infections worldwide has continued to climb, and the global COVID-19 epidemic continues to spread. In the discharged patients, many of them suffered from cardiopulmonary function and motor function decline due to the treatment of respiratory system and prolonged bed. After discharge, appropriate rehabilitation intervention should be carried out according to patients’ cardiopulmonary function, mental state and physical ability.^[18]^ Exercise therapy is a good intervention for skeletal muscle wasting and weakness.^[19]^ This paper will assess the prognostic impact of exercise therapy on COVID-19 patients, mainly including literature collection, literature screening, data extraction and data analysis. This study may provide some guidance for other researchers to study the prevention and treatment effect of exercise therapy on COVID-19 in the future. However, due to the limitation of language, we only searched Chinese and English literature, which is also the shortcoming of this study.

## Author contributions

**Conceptualization:** Guorong Qiu.

**Data curation:** Yajun Tan, Chunfeng Tan, Zhuolin Wang.

**Formal analysis:** Guorong Qiu, Yu Ji.

**Funding resources:** Yajun Tan.

**Investigation:** Chunfeng Tan, Zhuolin Wang, Hongpan Gao.

**Methodology:** Guorong Qiu, Yu Ji, Yajun Tan.

**Software:** Guorong Qiu, Benxiang He, Yu Ji.

**Writing – original draft:** Guorong Qiu.

**Writing – review & editing:** Benxiang He, Yu Ji.
